# Sibling-Support for Adolescent Girls (SSAGE): A study protocol for a pilot randomized-controlled trial of a whole-family, gender transformative approach to preventing mental illness among forcibly displaced adolescent girls

**DOI:** 10.1371/journal.pone.0303588

**Published:** 2024-05-31

**Authors:** Ilana Seff, Julianne Deitch, Arturo Harker Roa, Carolina Rodriguez, Tatiana Andia, Tamaity Ariza Pena, Lindsay Stark

**Affiliations:** 1 Brown School at Washington University in St. Louis, St. Louis, Missouri, United States of America; 2 Women’s Refugee Commission, New York, New York, United States of America; 3 Universidad de los Andes, Bogota, Colombia; 4 Mercy Corps, St, Portland, Oregon, United States of America; Public Library of Science, UNITED STATES

## Abstract

**Background:**

Forcibly displaced adolescents face increased risks for mental illness and distress, with adolescent girls disproportionately affected in part due to heightened gender inequity. Although the family unit has the potential to promote healthy development in adolescents, few family interventions have employed a gender transformative approach or included male siblings to maximize benefits for adolescent girls.

**Methods:**

This study will assess a whole-family and gender transformative intervention-Sibling Support for Adolescent Girls in Emergencies (SSAGE)-to prevent mental health disorders among adolescent girls in Colombia who were recently and forcibly displaced from Venezuela. The study will employ a hybrid type 1 effectiveness-implementation pilot randomized control trial (RCT) to test the program’s effectiveness to explore determinants of implementation to establish the feasibility, acceptability, and fidelity of SSAGE. To address these aims, we will enroll 180 recently arrived, forcibly displaced adolescent girls in an RCT and examine the program’s effectiveness in the prevention of mental illness (through reduction in anxiety, depression, interpersonal sensitivity, and somatization symptoms) one-month post-intervention. We will use contextually adapted to collect data on the hypothesized mechanistic pathways, including family attachment, gender-equitable family functioning, self-esteem, and coping strategies. The implementation evaluation will employ mixed methods to assess the program’s feasibility, acceptability, fidelity, and barriers and facilitators to successful implementation.

**Discussion:**

Findings can support humanitarian program implementation, as well as inform policy to support adolescent girls’ mental health and to prevent the myriad disorders that can arise as a result of exposure to displacement, conflict, and inequitable gender norms.

## Background

Forcibly displaced children and adolescents face heightened levels of mental distress and illness [1–3]. Evidence demonstrates that many experiences surrounding the forced migration process—such as exposure to conflict and violence, family loss or separation, and other adverse events—serve as risk factors for mental distress among displaced adolescents [2]. Adolescent girls face additional gendered exposures in humanitarian settings, including intimate partner and non-partner physical and sexual violence, early marriage, and sexual exploitation, which may serve to exacerbate mental distress and health [2,4]. Mental illness among forcibly displaced girls most often manifests through internalizing behaviors and symptoms, such as anxiety, depression, and psychosomatic symptomology [2,4,5].

The mental well-being of adolescent girls may be further impacted by gender inequitable attitudes and norms, which are often exacerbated in humanitarian settings [6–8]. Previous research from low-income country and refugee settings reveals a strong relationship between gender inequitable norms at the community level and lowered self-esteem and well-being at the individual level for adolescent girls [7,9]. Girls who internalize norms that marginalize and devalue their role within the family and community, may experience lowered self-esteem, perceived agency and resilience [7,10,11]. Inequitable household gender norms rooted in patriarchal systems rely not only on strict father-daughter interactions, but also on inequitable sibling relationships wherein adolescent male relatives such as brothers or cousins engage in disciplining girls for transgressing gender norms [12]. Further, fathers and brothers’ strategies for keeping girls safe in humanitarian settings often include limiting girls’ mobility, which can erode healthy coping and attachment and consequently increase vulnerability to mental health disorders when experiencing stressors [13,14].

Conversely, evidence shows that household environments characterized by high levels of family functioning, supportive relationships, and healthy caregiving practices, can promote resilience in children and adolescents [3,14,15]. For example, family connectedness was found to be inversely associated with internalized mental health symptoms among conflict-affected children and adolescents in Chechnya; and, in Gaza, supportive parenting practices were linked to reduced symptoms of depression, aggression, and other antisocial behavior among adolescents [15–17]. As such, family-level interventions have become an in increasingly employed approach for bolstering resilience and mental health among adolescents affected by conflict or experiencing forced displacement. The majority of literature supporting the utility of whole-family interventions in improving adolescent mental health derives from high-income countries, though a small body of evidence points to the promise of such interventions in low-income and humanitarian settings. A family-level psychosocial intervention implemented in the Democratic Republic of Congo, for example, reduce post-traumatic stress symptoms for youth or adolescents who had previously been abducted or had a family member who had been abducted [18,19]. However, a recent scoping review of whole-family interventions in humanitarian settings found only two programs employed a gender-transformative approach, and limited evidence speaks to the impact of no whole-family interventions in these settings incorporate gender-transformative approaches and limited evidence speaks to the potential consider gender-transformative considerations of whole-family interventions in these settings, nor considers, rigorous evidence is also lacking on effective family-based interventions that employ a gender-transformative approach to ensure positive impacts for adolescent girls, in particular [20].

To generate evidence on what works to prevent mental illness among forcibly displaced adolescent girls, Washington University in St. Louis, Universidad de Los Andes, Women’s Refugee Commission, and Mercy Corps are conducting a hybrid type 1 effectiveness-implementation pilot randomized-controlled trial (RCT) of Sibling Support for Adolescent Girls in Emergencies (SSAGE). SSAGE is a whole-family and gender-transformative mental health intervention that aims to foster more secure family attachments, improve girls’ self-esteem, and strengthen healthy coping skills on the path to improved mental health for forcibly displaced adolescent girls. The objectives of this hybrid type 1 pilot RCT are twofold. First, the study aims to examine the preliminary effectiveness and mechanistic pathways of SSAGE to prevent mental health disorders among recently arrived, forcibly displaced adolescent girls in Colombia; and second, the study seeks to assess the feasibility, acceptability and fidelity of the SSAGE intervention and identify determinants of successful implementation.

## Methods/Design

We will undertake a hybrid type 1 effectiveness-implementation pilot RCT to test the effectiveness and mediators of SSAGE while simultaneously using a concurrent, convergent mixed methods design to examine the acceptability, feasibility, and fidelity of the intervention.

### Study setting

Venezuela, once one of the wealthiest countries in Latin America, has faced acute economic and social collapse in the past several decades [21]. State instability, hyperinflation, plunging access to food and medicine, and high increases in crime rate [22,23] have had profound impacts on Venezuelans and led to the second-largest external displacement crisis in the world [24]. Among the more than 6 million Venezuelans who have migrated out of the country as of February 2022, [22,24,25] nearly five million are forced migrants who have fled to neighboring countries such as Colombia, Ecuador, and Peru; [22,24,25] as of August 2021, Colombia hosts the vast majority of Venezuelan refugees and migrants [24,26]. In addition to increasing numbers of migrants who have left Venezuela with the intention of remaining in Colombia [26], the current migratory wave has seen an uptick in Colombian returnees and pendular migrants, or Venezuelans who transit between Colombia and Venezuela daily [22,27].

Colombia has taken action to support the Venezuelan diaspora entering the country, including the creation of Special Stay permits that allow Venezuelans to reside in the country for at least two years.[22] Additionally, Colombia enacted a policy in 2021 to provide displaced Venezuelans a temporary protection status for the next 10 years.[22] The majority of Venezuelan migrants and Colombian returnees from Venezuela are concentrated in the north-eastern regions of Colombia, where Cartagena—the study setting—is located, largely due to the ease of transit between the countries [28]. Venezuelan migrants primarily arrive in areas already affected by armed conflict, higher levels of multidimensional poverty, and strong social norms and beliefs that normalize gender stereotypes and GBV [29].

The city of Cartagena is located in Colombia’s Bolívar department, which receives among the largest number of females who are forcibly displaced from Venezuela, including Colombian returnees. The proximity of the department to the Colombian-Venezuelan border and its cultural dynamics make Cartagena the first city within the department to house persons who are forcibly displaced from Venezuela. Further, operating in a larger metropolitan area will ensure access to hospitals for severe mental health referrals, if needed.

### Intervention description

SSAGE is a gender-transformative, 12-week program utilizing a “whole family approach” wherein an adolescent girl, her male sibling, and a male and female caregiver participate in sessions that are age- and gender-specific and combined with family-wide discussions of session learnings [30]. The sessions are interactive, engaging, and promote self-reflection and discussion on topics such as power, gender, interpersonal communication, and healthy relationships. Given the whole-family approach, SSAGE addresses intersections between spousal relationships, caregiver-child relationships, and relationships between siblings, as they pertain to supporting the mental health and psychosocial well-being of adolescent girls [31]. The SSAGE program improves family functioning by engaging the entire family unit in discussion; adolescent boys receive normative support from other boys and adolescent girls receive support from their peers, thereby improving social support in a manner that is sensitive to gender norms.

**Fig 1:**
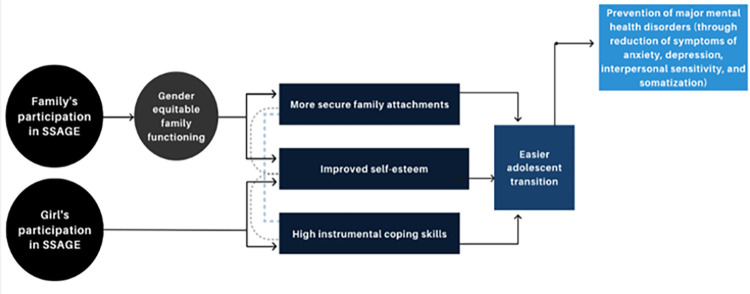
Conceptual model for SSAGE. Source: [32].

As shown in Fig 1, SSAGE is hypothesized to prevent mental health disorders among adolescent girls through two pathways of action (direct and indirect). For the direct pathway, adolescent girls’ own participation in SSAGE activities (i.e., life skills curricula, girl-friendly community spaces, sessions on bodily knowledge and autonomy, etc.) will strengthen instrumental coping skills and self-esteem. Girls’ confidence in applying problem-focused strategies to mitigate stressors related to their adolescent development is conducive with a healthy adolescent transition and will reduce symptoms of depression, anxiety, and distress, thereby preventing the later development of major mental health disorders. For the indirect pathway, parents/caregivers and older male siblings’ (or other male adolescent relatives’) participation in SSAGE activities (i.e., skills building for healthy parenting, understanding adolescent girls, gender socialization, etc.) will promote gender equitable family functioning, which in turn will support more secure family attachments and adolescent girls’ self- esteem. Feeling supported by family members and included in household decision making will reduce the impact that adolescence-relate stressors have on the development on anxiety, depression, and distress symptoms and later disorders among adolescent girls.

SSAGE has previously been implemented in Northeast Nigeria and with Syrian refugees in Jordan, and has shown promise in promoting family functioning, gender equity, and healthy masculinity among family members living in Northeast Nigeria and refugee camps in Jordan. Qualitative data collected from participants in Nigeria revealed key ways in which the program improved elements of family functioning; participants reported more equitable gender roles and attitudes such as joint decision making among caregivers, men and boys’ participation in household labor, reduced stigma when discussing menstruation and puberty and a reduction in intrahousehold violence against girls [30,31]. Additional positive impacts for adolescent girls were demonstrated in Jordan, where girl participants exhibited higher levels of mental well-being, resilience, and perceived family functioning [32]. Although previous implementation experiences highlight positive reflections for participants, this study will assess SSAGE’s impact on mental health through the mechanisms outlined in Fig 1 and identify potential barriers to implementation that may circumscribe the program’s intended effects. The SSAGE curriculum will be culturally adapted through a human-centered design process prior to the evaluation [33,34].

### Study population, recruitment, and retention

Recruitment for this study will draw from families who fled Venezuela and relocated to Colombia within the last year. Included among this population are families originally born in Colombia who had previously emigrated to Venezuela for at least five years and subsequently returned to Colombia. Eligibility requirements include adolescent girls aged 13–19 years old who: live with a male and female caregiver and an adolescent male sibling or relative; immigrated to Colombia within the last year; are available, along with their family members, to participate in the SSAGE intervention for three months; and are available to participate in survey questionnaires immediately before and one month after the intervention (please see Fig 1 for a study schedule). We will recruit 180 adolescent girls from 180 families. Study participants will be recruited by Mercy Corps and consented by Los Andes University and Washington University (Table 1).

**Table 1:** Schedule of enrolment, interventions, and assessments.

	STUDY PERIOD					
	Enrolment		Post-allocation			Close-out
TIMEPOINT	09/24-10/24	11/24	12/24	01/25-03/25	04/25	08/25
**ENROLMENT:**						
Eligibility screen	X					
Informed consent	X					
Allocation		X				
**INTERVENTIONS:**						
Treatment: SSAGE				X		
Control: Care as usual				X		
**ASSESSMENTS:**						
Baseline: Mental illness, anxiety and depression			X			
Endline: Mental illness, anxiety and depression					X	
Gender equitable family functioning, family attachment, self-esteem, instrumental coping skills			X		X	
**CLOSE-OUT:**						X

Following recruitment into the study, adolescent girl participants and their families will be tracked by program facilitators to record retention using attendance data. Each adolescent girl participant will be assigned a unique study identification number to enable tracking survey responses across points of data collection. Successful retention in the study depends almost entirely on retention in the SSAGE program itself. Mercy Corps Colombia employs several strategies with its programming to maximize participant retention. First, Mercy Corps guarantees that all programming takes place in a safe space, defined as one in which all voices are heard without judgment, the diversity of participant experiences and mental states is recognized and respected, participants do not develop false expectations, and the space offers a reprieve from the distress inherent in everyday life. Second, Mercy Corps coordinates with local schools to schedule adolescent programming during school to avoid an added time burden for adolescent participants. Third, given that transportation costs can be a financial barrier for this population, Mercy Corps provides transportation resources to ensure participants can attend sessions without having to allocate their own resources. Finally, the schedule for program sessions will be determined collaboratively between participants and program staff in order to prioritize availability and maximize convenience. These strategies have proved successful in retaining participants for past Mercy Corps programs. The research team will also employ the same strategies for data collection sessions.

### Study timeline, data collection and outcomes of interest

Following the recruitment of 180 girls from 180 families, 90 adolescent girls each will be randomized to the treatment and control arm using a random number generator. Randomization will not be blinded. To measure effectiveness of the intervention, all 180 adolescent girls in the study (both treatment and control arms) will complete a survey questionnaire through an enumerator-facilitated interview using computer-assisted personal interview (CAPI) software immediately prior to the onset of the intervention. These same participants will be visited one month following the completion of the 12-week intervention (T2) to take the same survey using CAPI. Study participants’ random study identification will be used to link their survey responses between baseline and T2.

The primary outcome measures include mental illness, anxiety and depression. A full list of primary and secondary outcomes, corresponding sources of measurement, and hypothesized directions of change can be found in Table 2. Relevant socio-demographic variables will also be collected, including age, education status, caregiver employment status, current living situation and household composition, relationship status, time in Colombia, country of birth, stressful life events, and other support services being received by the household. Survey tools will be translated into Spanish and back-translated.

**Table 2:** Primary and secondary outcomes and hypothesized direction of change.

Construct as presented in Fig 1	Source of measure	Hypothesized direction of change
**Primary outcomes of interest**		
Mental illness	DSM-V cross-cutting youth [35]	Decrease
Anxiety and depression	Revised Child Anxiety and Depression Scale-25 [36]	Decrease
**Secondary outcomes/mediators**		
Gender equitable family functioning	Gendered responsibilities scale [37]	Increase
Family attachment	Family Attachment and Changeability Index (FACI8) [38]	Increase
Improved self-esteem	Rosenberg Self-Esteem Scale (RSES) [39]	Increase
High instrumental coping skills	Kidcope [40]	Increase

Before each participant leaves the data collection site, their data will be reviewed by a data collector for completion. All data will be incrementally backed up each day on a secure data system and a full back-up will be performed weekly. The research team at Washington University in St. Louis will be responsible for weekly data checks to ensure data quality; errors will be reported to the local field team and rectified. All data collected during the pre- and post-assessments will be stored on secure tablets provided by University of Los Andes. Only selected, authorized WRC, Mercy Corps, University of Los Andes, and Washington University staff will be able to unlock the password-protected tablets.

### Sample size and power analysis

To detect a medium effect size in our primary outcomes of interest of d = 0.5, assuming statistical power of 80% and a one-sided alpha of 0.05, we need a sample size of 63 adolescent girls in each study arm. We expect approximately 20% attrition from baseline to T2 either through: (1) declining to participate in subsequent rounds of data collection or (2) data collectors’ inability to locate the participant. Additionally, when estimating the effect of SSAGE on the four outcomes of interest, we will exclude adolescent girls already exhibiting above-threshold scores on the four-dimension measure of mental health symptoms at baseline as it would not be possible to ‘prevent’ mental health disorders in this subgroup. We expect this to result in the loss of an additional 10% of the initial sample. As such, we require a sample size of 90 girls for each study arm.

### Statistical methods

To assess the effectiveness of SSAGE in preventing mental health disorders, we will test the null hypothesis that adolescent girls who participate in SSAGE exhibit the same or greater levels of mental health symptoms as compared to girls who receive care as usual. We will test this hypothesis using both an intent-to-treat and per protocol analytical approach [41,42]. We will employ generalized linear regression models to estimate the effect of the intervention on the primary outcomes, controlling for covariates not balanced between the two study arms at baseline; given that treatment assignment is random, we expect that we will not need to control for many covariates. Per protocol analysis will also be conducted, leveraging attendance data from all participating family members. The threshold for meeting program adherence will be defined alongside program implementers prior to program implementation.

As a secondary, exploratory analysis, we will examine the effect of SSAGE on gender equitable family functioning, family attachment, self-esteem and coping strategies (see mechanistic pathways in Fig 1); and, we will assess if the effect of SSAGE on each of the primary outcomes of interest is mediated by these four secondary measures. The effect of SSAGE on each secondary outcome will be assessed using linear regression models (as described above). Mediation analysis will be conducted using structural equation modeling to estimate the degree to which each secondary measure mediates the impact of SSAGE on the prevention of mental health disorders.

To avoid a reduction in statistical power and potentially biased results, we will employ iterative Monte Carlo Markov Chain (MCMC) multiple imputation to handle missing data in Stata. We will first generate 50–100 imputed datasets using expectation-maximization algorithm as estimates on which we will employ the MCM procedure. All statistical inferences will then be made using the combined results across all complete datasets

### Implementation evaluation

Qualitative implementation evaluation data–including key informant interviews with program staff and mentors (n = 16) [43] and semi-structured in-depth interviews with a subset of individuals in the treatment arm (n = 20)–will be conducted immediately following the end of the intervention to assess program acceptability, feasibility, and fidelity, and to identify implementation determinants (i.e., barriers and facilitators) that may influence the quality of program delivery.[44,45] Program participants will be purposively sampled in order to ensure representation from all cohorts (male caregivers, female caregivers, adolescent boys, and adolescent girls) as well as levels of adherence to the treatment protocol including number and length of sessions delivered as well as attendance. By also including participants with low attendance, we will be able to explore fidelity as well as potential barriers to acceptance and feasibility. Program mentors and staff will also be purposively sampled to ensure representation across target cohorts and levels of oversight, respectively, in order to inform an exploration of implementation determinants. Interview guides will be guided by the Exploration, Preparation, Implementation, and Sustainment (EPIS) Framework [46,47] and Implementation Outcomes Framework [48] and will focus on the facilitators of and barriers to program implementation as well as the acceptability, feasibility, and fidelity of SSAGE. Interviews will be recorded, translated, transcribed, and cleaned for analysis.

SSAGE’s acceptability and feasibility will also be measured through administration of the acceptability and feasibility sub-scales of the Mental Health Implementation Science Tools (mhIST). These two scales will be administered to all treatment participants (n = 90) at T2, as part of the quantitative survey [49]. The mhIST was developed specifically for use in low-resource settings and the tool has been validated in multiple low- and middle-income countries, including in Colombia [49]. Each scale includes 15 questions that are answered by the respondent using a Likert scale.

Descriptive statistics for the mhIST items and scales will first be estimated to identify perceptions of program acceptability and feasibility. Qualitative data will be imported into a qualitative data analysis software and analyzed using qualitative content analysis, a theory-driven approach that will involve both deductive and inductive coding [50,51]. Deductive codes will be derived from guiding conceptual frameworks [46,48,52]. Mixed methods analyses will involve merging the quantitative and qualitative data in joint displays to examine the extent to which the two types of data converge. These results will inform further refinements of SSAGE and the identification of implementation strategies that may be needed to address identified determinants and ensure that the intervention is routinely delivered with high quality.

## Discussion

All study protocols were approved by the Washington University in St. Louis and Los Andes University Institutional Review Boards. Following screening, research team members will obtain and document voluntary and informed consent from all adolescent girl participants aged 18 and over and informed assent from all participants under the age of 18, following their caregivers’ consent. Researchers collecting consent and assent will read adolescent girls and/or their participating family members a standardized document explaining the voluntary nature of the study, as well as the benefits and risks of participation. After addressing participants’ and/or caregivers’ questions or concerns, the enumerator will request that the participant sign, fingerprint, or otherwise mark the consent form to indicate agreement to participate in the study. Researchers will obtain consent from one caregiver on behalf of each adolescent participant under the age of 18 before researchers obtain assent from adolescents whose caregivers have consented on their behalf. All surveys will be administered in a private area and all enumerators will be female to maximize the comfort of adolescent girl participants.

Given the potentially sensitive nature of topics discussed during program sessions, all research staff will complete training on the importance of ensuring privacy and confidentiality. There is also the possibility for negative emotional or psychological responses while completing the survey measures depending on the nature of participants’ experiences of violence, conflict, displacement, and prior mental health challenges. Study procedures will include voluntary referral mechanisms available to connect distressed participants with follow-up support, as needed. If any participants express distress during the data collection, enumerators will be required to fill out an adverse event form to report the event and the local team’s response, including ay referral pathways offered. All forms will be collected and reported by Mercy Corps to the study investigators. However, risks to study participants are minimal and, given the pilot nature of the trial, a data monitoring committee will not be convened [53].

This study concept was approved by the Washington University in St. Louis Institutional Review Board (#202304121). All protocol modifications will be reported to this review board and the clinical government trial registry.

This hybrid type 1 effectiveness-implementation pilot RCT will offer insights on how to support adolescent girls’ mental health and prevent the myriad disorders that can arise as a result of exposure to displacement, conflict, and inequitable gender norms in their households and communities. While this evaluation will be focused on evaluating SSAGE’s implementation and effectiveness in mitigating adverse mental health outcomes for adolescent girls, SSAGE is well positioned to impact a number of other domains which may also contribute to girls’ mental health and well-being. In Nigeria, for example, SSAGE was found to improve household communication around sensitive health topics such as puberty, menstruation, and experiences of GBV [30]. The decision to adapt SSAGE in the Colombian context increases the program’s potential to improve the health of one of the largest displaced populations globally. Migration patterns indicate that Peru, Chile, Ecuador, Brazil, Argentina, Panama, and Mexico are among the top Latin American countries that receive forcibly displaced Venezuelans. The cultural and contextual adaptations that will be made to the existing SSAGE program will also be relevant for Venezuelan adolescent girls who are forcibly displaced in these other Latin American countries. Finally, evaluating this implementation process will produce knowledge for how to effectively generate greater humanitarian program impact when SSAGE is implemented with similar populations within the U.S., as well as with other populations experiencing displacement around the globe.

## Supporting information

S1 Checklist SPIRIT 2013 checklist: Recommended items to address in a clinical trial protocol and related documents*. (DOC)

S1 File (DOCX)
